# Predictors of the Efficacy of Lymphedema Decongestive Therapy

**DOI:** 10.3390/medicina61020231

**Published:** 2025-01-27

**Authors:** Andrej Dzupina, Nagendra Yaluri, Jaipaul Singh, Monika Jankajova

**Affiliations:** 1 Department of Angiology, National Heart Institute, 833 48 Bratislava, Slovakia; a.dzupina@gmail.com; 2 Centre of Clinical and Preclinical Research MEDIPARK, Pavol Jozef Safarik University, 040 01 Kosice, Slovakia; 3 School of Pharmacy and Biomedical Sciences, University of Central Lancashire, Preston PR1 2HE, UK; jaipsingh10@gmail.com; 4 Cardiology Department, Kardiocentrum Agel-Saca a.s., Lúčna 57/040 15, 040 15 Kosice, Slovakia

**Keywords:** lymphedema, decongestive therapy, diagnosis, vascular, skin, angiology

## Abstract

Lymphedema is a chronic condition characterized by the accumulation of lymphatic fluid in the tissues, causing swelling primarily in the limbs, though other body parts can also be affected. It commonly develops after lymph node removal, or radiation therapy, or due to congenital lymphatic system defects. Effective management is essential due to its significant impact on physical function and quality of life. Complete Decongestive Therapy (CDT) is the primary treatment for lymphedema. This comprehensive approach combines manual lymphatic drainage (MLD), compression bandaging, skincare, and exercise. An early diagnosis and initiation of CDT are critical to preventing irreversible damage to the lymphatic system and worsening symptoms. Successful outcomes depend on timely treatment, patient adherence, and the consistent use of all CDT components, with compression therapy and exercise playing particularly vital roles. Recent research highlights how skin and fat tissue characteristics, such as increased skin thickness and adipose tissue accumulation, complicate lymphedema management, especially in advanced stages. In these cases, where fibrosis and fat deposition are more prominent, traditional CDT may need to be supplemented with advanced treatments like liposuction or enhanced compression techniques. This study explores the factors influencing the success of decongestive therapy, including the stage of lymphedema at the diagnosis, treatment protocols, and individual patient characteristics like skin and fat tissue properties.

## 1. Introduction

### 1.1. Pathophysiology of Lymphedema

The lymphatic system plays a crucial role in maintaining fluid balance by filtering plasma containing proteins, water, and waste products from interstitial spaces and returning it to the bloodstream. In a healthy system, this process works efficiently [1]. However, disruptions in the lymphatic network can lead to the accumulation of lymph fluid in tissues, causing edema. Lymphedema can result from various factors, including changes in capillary or osmotic pressure, muscle integrity issues such as fibrosis or reduced muscle tone, and damage to lymph nodes caused by chemotherapy, radiation, or surgical removal. Other causes include congenital abnormalities of the lymphatic system, severe skin trauma, or infections. When lymph flow is obstructed, fluid builds up in the tissues because proteins and debris cannot return to the bloodstream effectively. This swelling, known as lymphedema, can occur in any part of the body where lymphatic drainage is compromised [1,2]. The diagnosis of lymphedema carries numerous psychological, physical, and social implications. It is broadly categorized as either primary, with a genetic basis, or secondary, in which the patients acquired the condition through external factors [2].

The function of the lymphatic system is to transport lymph, a fluid containing white blood cells, triglycerides, bacteria, cell debris, water, and protein, akin to blood plasma. This intricate drainage system comprises initial lymphatics (lymph capillaries), pre-collectors, collectors, lymphatic trunks, and lymph nodes [3]. Topographically, the lymph system is divided into superficial (subcutaneous) and deep (subfascial) components. The superficial system drains the skin and subcutaneous tissues, while the deep system handles muscles, joints, tendon sheaths, and nerves. Perforating vessels serve as connections between these systems, facilitating the transport of lymph from subfascial areas to the surface [4,5].

Signs and symptoms of lymphedema encompass distal swelling in extremities such as the arms, hands, legs, and feet, as well as proximal swelling in areas like the breast, chest, shoulder, pelvis, groin, genitals, face, and intraoral tissues. Restricted range of motion in joints due to swelling and tissue changes, skin discoloration, pain, altered sensation, limb heaviness, and difficulty in fitting into clothing are also common manifestations [6,7,8].

### 1.2. Decompressive Therapy in Lymphedema

Decompressive therapy in lymphedema is the gold standard treatment approach aimed at reducing swelling and managing the symptoms associated with lymphedema, which is a condition characterized by the buildup of lymph fluid in the tissues of the body, typically due to a blockage in the lymphatic system. Decompressive therapy encompasses various techniques and modalities designed to alleviate swelling and improve lymphatic circulation [9,10]. Some common methods of decompressive therapy in lymphedema include the following.

### 1.3. Manual Lymphatic Drainage (MLD)

This is a gentle massage technique performed by a trained therapist to stimulate the flow of lymph fluid and encourage its drainage from the affected area [11,12]. Manual lymphatic drainage (MLD) is a specialized massage technique commonly used in the management of lymphedema. It involves gentle, rhythmic movements that stimulate the lymphatic system, promoting the drainage of excess lymphatic fluid and reducing swelling in affected areas. MLD is often performed by trained therapists who apply specific hand movements to redirect lymph fluid towards functional lymphatic pathways. Several studies have demonstrated the effectiveness of manual lymphatic drainage in the treatment of lymphedema. A study published by Koul et al. [13] assessed the impact of MLD on breast cancer-related lymphedema. The results showed significant reductions in limb volume and improvements in symptoms following MLD treatment. A systematic review and meta-analysis were conducted by Ezzo et al. [12], who evaluated the efficacy of MLD in the management of lymphedema. The analysis included several randomized controlled trials and concluded that MLD was effective in reducing limb volume and improving quality of life in patients with lymphedema. Another study by Devoogdt et al. [14] published in *Cancer Nursing* investigated the long-term effects of MLD on breast cancer-related lymphedema. The findings indicated sustained improvements in limb volume and symptom relief over a 12-month follow-up period. These studies highlight the therapeutic benefits of manual lymphatic drainage in reducing swelling and improving symptoms associated with lymphedema. However, it is important to note that MLD is often used as part of a comprehensive treatment approach, which may include compression therapy, exercise, skincare, and patient education.

### 1.4. Compression Therapy

Compression garments or bandages are applied to the affected limb to help in reducing swelling by applying external pressure and supporting the lymphatic vessels [15,16,17]. Compression therapy is a cornerstone of treatment for lymphedema, aimed at reducing swelling and improving lymphatic function through the application of external pressure. It typically involves the use of compression garments, bandages, or pneumatic compression devices to exert pressure on the affected limb or body part. Compression therapy helps in promoting lymphatic drainage, preventing fluid accumulating, and maintaining the reduced size of the limb and this is achieved through other treatments such as manual lymphatic drainage. Several studies have investigated the efficacy of compression therapy in the management of lymphedema. A randomized controlled trial by Ochalek et al. [18] compared the effectiveness of different compression garments in reducing arm lymphedema following breast cancer treatment. The study found that compression therapy significantly reduced limb volume and improved symptoms compared to no treatment. A systematic review and meta-analysis by McNeely et al. [19] evaluated the evidence for compression therapy in breast cancer-related lymphedema. The analysis included several studies and concluded that compression garments were effective in reducing limb volume and improving quality of life in patients with lymphedema. A study by Mayrovitz et al. [20] investigated the effects of intermittent pneumatic compression on lower-extremity lymphedema. The results demonstrated significant reductions in limb volume and improvements in tissue fluid content following pneumatic compression therapy. These studies provide evidence to support the use of compression therapy as an effective intervention for managing lymphedema. It is important to note that the selection and application of compression garments or devices should be tailored to the individual needs of the patient and preferences and moreover, it should be properly monitored since it is essential to ensure optimal outcomes.

### 1.5. Exercise

Specific exercises and movements of the body may be prescribed to promote lymphatic flow and to improve muscle pumping action, which can aid in reducing swelling [21,22]. Exercise plays a crucial role in the management of lymphedema by promoting lymphatic flow, improving muscle pump function, and enhancing overall physical and mental well-being of the patient. However, it is important that exercise programs are tailored to individual capabilities and carefully monitored to prevent injury and the exacerbation of lymphedema symptoms. Here are some key points about exercise and lymphedema.

#### 1.5.1. Benefits of Exercise

Exercise can help to reduce swelling, improve range of motion, increase strength and flexibility, and enhance overall quality of life for individuals with lymphedema. It also promotes cardiovascular health and aids in weight management, which are important considerations for lymphedema management.

#### 1.5.2. Types of Exercise

Low-impact aerobic exercises such as walking, swimming, cycling, and aquatic exercises are generally safe and effective for individuals with lymphedema. Resistance training and flexibility exercises can also be beneficial, but should be performed with caution and under supervision, particularly for individuals at risk of developing or exacerbating lymphedema.

#### 1.5.3. Precautions and Guidelines

It is important for individuals with lymphedema to follow specific precautions and guidelines when exercising. These include wearing compression garments during activity, avoiding repetitive or high-impact movements that may strain the affected limb, and gradually increasing exercise intensity and duration under the guidance of a healthcare professional.

Several studies have investigated the effects of exercise on lymphedema management. A randomized controlled trial by Ahmed et al. [23] evaluated the effects of supervised aerobic and resistance exercise in breast cancer survivors with lymphedema. The study found that exercise led to significant reductions in arm swelling and improvements in physical function and quality of life. A systematic review by Cheema et al. [24] published in *Breast Cancer Research and Treatment* examined the effects of exercise on lymphedema-related outcomes in breast cancer survivors. The review concluded that exercise was safe and beneficial for individuals with lymphedema, leading to improvements in limb volume, physical function, and psychosocial well-being. Another study by Czerniec et al. [25], published in *Breast Cancer Research and Treatment*, investigated the effects of resistance training on breast cancer-related lymphedema. The study found that resistance training led to improvements in arm volume and strength without exacerbating lymphedema symptoms. These studies provide strong evidence supporting the inclusion of exercise as part of a comprehensive lymphedema management program. However, it is necessary for individuals with lymphedema to consult with their healthcare providers before starting an exercise regimen and to receive guidance on safe and appropriate activities based on their individual needs and health status.

### 1.6. Pneumatic Compression Therapy (PCT)

This involves the use of pneumatic compression devices that intermittently inflate and deflate sleeves or boots worn on the affected limb, promoting lymphatic drainage [15,26]. PCT is a non-invasive treatment modality used in the management of lymphedema. It involves the application of sequential or intermittent pneumatic compression devices to the affected limb or body part, which exert external pressure and promote lymphatic drainage. PCT works by enhancing tissue fluid movement, reducing swelling, and improving lymphatic function. PCT applies external pressure via compression garments to the affected limb through the sequential or intermittent inflation and deflation of air chambers within the compression device. This action helps to mimic the natural pumping action of muscles and promotes the movement of lymphatic fluid towards functional lymphatic pathways, facilitating drainage and reducing swelling. Several studies have demonstrated the effectiveness of PCT in the management of lymphedema. A randomized controlled trial by Nelson at al. [27] investigated the effects of intermittent pneumatic compression on lower-extremity lymphedema. The study found significant reductions in limb volume and improvements in tissue fluid content following PCT. A systematic review and meta-analysis by Uzkeser et al. [28] evaluated the efficacy of PCT in the treatment of lymphedema. The analysis included several studies and concluded that PCT was effective in reducing limb volume and improving symptoms in individuals with lymphedema.

PCT is recommended as a treatment option for lymphedema in clinical practice guidelines, including those from organizations such as the National Lymphedema Network (NLN) and the International Society of Lymphology (ISL) [2,29]. These guidelines emphasize the importance of using PCT as part of a comprehensive lymphedema management program, which may include other modalities such as manual lymphatic drainage, compression garments, exercise, and skincare [2,29,30]. PCT is generally well tolerated by patients with lymphedema, but it is essential to consider individual factors such as comfort, mobility, and treatment preferences when prescribing PCT. Proper fitting of compression garments and regular monitoring of treatment progress are important aspects of patient care [31,32,33].

### 1.7. Complete Decongestive Therapy (CDT)

CDT is a comprehensive treatment program for lymphedema that typically includes a combination of manual lymphatic drainage, compression therapy, exercise, skincare, and patient education [34,35]. CDT is a comprehensive treatment approach commonly used in the management of lymphedema. It consists of several components, including manual lymphatic drainage (MLD), compression therapy, exercise, skincare, and patient education. CDT aims to reduce swelling, improve lymphatic function, and enhance the overall quality of life for individuals with lymphedema. Here are some key points about CDT and lymphedema, along with references supporting its efficacy. A randomized controlled trial by Stout Gergich et al. [36,37] published in *Cancer* assessed the effectiveness of CDT in breast cancer-related lymphedema. The study found significant reductions in limb volume and improvements in symptoms following CDT treatment. A systematic review by Stout et al. [38] evaluated the evidence for CDT in the management of lymphedema. The review concluded that CDT was effective in reducing limb volume, improving quality of life, and minimizing complications associated with lymphedema. CDT is recommended as the standard of care for lymphedema management in clinical practice guidelines, including those from organizations such as the National Lymphedema Network (NLN) and the International Society of Lymphology (ISL). These guidelines emphasize the importance of a multidisciplinary approach and the integration of various components of CDT for optimal treatment outcomes. Individualized assessment and treatment planning are essential in CDT to tailor interventions to each patient’s specific needs, preferences, and clinical presentation. Regular monitoring and adjustment of treatment regimens are also important to ensure ongoing effectiveness.

### 1.8. Surgical Options

Surgical options are available for the management of lymphedema, particularly in cases where conservative treatments such as CDT have not provided sufficient relief or when the condition is severe. Some surgical interventions can be performed in an outpatient setting and the key for a successful outcome is time planning, whereas the surgery should be planned after the intensive phase of CDT. Surgical interventions are generally aimed to improve lymphatic flow, reduce swelling, and alleviate symptoms. Surgical treatments for lymphedema are categorized into two main types. They are Reductive Methods and Pfhysiologic Methods. Reductive Methods involve the removal of lymphedematous tissue, such as extensive lipectomy or excisional surgeries like the Charles procedure and radical reduction with the preservation of perforators (RRPP). Liposuction is commonly used to reduce localized fat deposits and reshape body contours. It involves the suction removal of fat from lamellar deposits using a cannula connected to a vacuum source, which is carefully passed through fatty tissue [39,40,41]. The Charles procedure is one of the oldest treatments for lymphedema, involving the excision of affected tissue down to the fascia and skin grafting. Removed tissue may be repurposed as skin grafts to cover the surgical site. Recovery can take up to a month, with extensive postoperative care required. In severe cases where subcutaneous tissue becomes too fibrotic for liposuction, excisional surgeries like RRPP or a modified Charles procedure are necessary to significantly reduce lymphatic load. A modified Charles procedure involves removing soft tissue through a long incision in the affected area, sometimes including skin removal and grafting if the skin is damaged. This complex surgery typically requires a 3–5-day hospital stay, followed by possible long-term care for full recovery [40,42]. Radical reduction with perforator preservation (RRPP) is an advanced technique combining traditional excision with microsurgical principles to remove diseased tissue while maintaining blood supply to the overlying tissue through perforator vessels [40]. The Physiologic Methods focus on restoring lymphatic function, including vascularized lymph node transfer (VLNT) and lymphaticovenular anastomosis (LVA). VLNT involves transplanting functional lymph nodes to restore lymphatic flow. Microanastomosis is performed to connect the transplanted nodes to the vasculature at the recipient site, ensuring that their blood supply is maintained. LVA redirects excess lymphatic fluid into the venous system. By connecting lymphatic vessels to nearby veins, this procedure reduces lymphedema and helps to prevent its recurrence, allowing lymph to drain back into systemic circulation [43,44]. In some cases, a combination of surgical techniques, such as LVA and VLNT or VLNT and liposuction, may be used to optimize outcomes and address multiple aspects of lymphedema pathophysiology. A study by Cheng et al. [45] evaluated the effectiveness of combined surgical approaches in the management of lymphedema. The study demonstrated favorable outcomes in terms of volume reduction and symptom improvement.

## 2. Predictors of Success of Decompressive Therapy of Lymphedema

In a study published by Can et al. [46], they reported that the predictors of successful outcomes in decompressive therapy for lymphedema include patient education, skincare, regular exercise, and the implementation of compression therapy. The study also identified factors such as advanced age, obesity, larger tumor size, a higher number of positive lymph nodes, and postoperative radiotherapy as risk factors that were associated with lymphedema, suggesting that these could also influence the effectiveness of decompressive therapy [46]. In a preliminary study by Kwon et al. [47], the authors highlighted the predictive value of preoperative lymphoscintigraphy for determining the success of lympho-venous anastomosis in treating lymphedema. Lymphoscintigraphic indicators such as the dermal backflow pattern and extremity uptake ratio can predict both early and late therapy responses, essential for planning effective decompressive therapy [47]. Lemoine et al. [48] reported that effective decompressive therapy in the immediate postoperative period, especially in acute settings such as after head and neck surgery, can significantly reduce lymphedema volumes. This study demonstrates that early intervention with decongestive therapy is feasible and effective, serving as a predictor of successful lymphedema management [48]. The above evidence collectively identifies early intervention, patient adherence to recommended therapies, and the use of diagnostic tools like lymphoscintigraphy as key predictors of successful outcomes in decompressive therapy for lymphedema. Factors such as the patient’s age, tumor characteristics, and the presence of obesity also play a significant role in determining the effectiveness of therapy.

Additionally, in a paper by Shallwani et al. [49], the authors confirm the importance of tailored interventions, including compression therapy and individualized exercise, in predicting successful outcomes in the management of lymphedema in women treated for gynecological cancer. The feasibility of implementing these interventions suggested that a multidimensional approach could be crucial in managing lymphedema effectively [49]. In a study conducted by Lanza et al. [50], they show that treatment protocols such as compression bandaging and manual lymph drainage are effective predictors of successful volume reduction in lymphedema. However, the frequency of treatments and adherence to protocols established at each service significantly impact the therapeutic response [50]. In a research paper by Kwon et al. [47], they highlight the predictive role of preoperative lymphoscintigraphy in determining the success of lympho-venous anastomosis for lymphedema treatment. The dermal backflow pattern and extremity uptake ratio serve as crucial indicators for predicting both early and late therapy responses [47]. Finally, a study conducted by Borman et al., [51], underscores the importance of the number of dissected lymph nodes as a key factor influencing the development and management of lymphedema. It suggests that understanding the extent of surgical intervention can help predict and manage the risk of lymphedema more effectively [51]. These four studies outlined above provide additional evidence supporting the importance of individualized treatment plans, early diagnostic interventions, and understanding surgical impacts as key predictors in the successful management of lymphedema through decompressive therapy (Table 1).

## 3. Additional Predictors of Success of Decompressive Therapy of Lymphedema

### 3.1. Stage and Severity of Lymphedema

The stage and severity of lymphedema are crucial predictors of treatment outcomes and play a significant role in guiding therapeutic interventions. Lymphedema staging refers to the classification of the condition based on its progression and clinical presentation, while severity refers to the extent of tissue swelling and functional impairment. Understanding these factors is essential for developing effective treatment plans and predicting response to therapy. The International Society of Lymphology (ISL) provides guidelines for the diagnosis and treatment of peripheral lymphedema, emphasizing the importance of accurate staging in treatment planning [52]. Early-stage lymphedema, characterized by mild swelling and minimal tissue changes, may respond more favorably to treatment compared to advanced-stage lymphedema where tissue fibrosis and irreversible changes have occurred [53]. In addition to staging, the severity of lymphedema also influences treatment outcomes. Szuba et al. [54] discuss the progressive nature of lymphedema and its impact on tissue integrity, emphasizing the need for early intervention to prevent irreversible damage. Damstra et al. [55] compare different compression therapy modalities for arm lymphedema and highlight the role of treatment intensity in managing lymphedema severity. Overall, accurate assessment of the lymphedema stage and severity is essential for predicting treatment outcomes and tailoring interventions to individual patient needs. Early intervention and appropriate management strategies can help optimize outcomes and improve the quality of life for individuals living with lymphedema.

### 3.2. Extent of Fibrosis

The extent of fibrosis in lymphedematous tissue serves as an important predictor for treatment outcomes and can significantly influence the efficacy of therapeutic interventions. Fibrosis, the thickening and scarring of tissue from pathological repair, often stems from injury but can also result from metabolic changes in adipose tissue that trigger inflammation. Adipose tissue fibrosis is typically undetectable, difficult to reverse, and complicates the treatment of obesity and related conditions. Increased adipocyte size and tissue mass promote inflammation, disrupting normal adipose tissue function and leading to fibrosis. Unlike healthy adipose tissue, which can remodel its extracellular matrix (ECM) to adapt to size changes, fibrotic tissue becomes stiff and loses this ability. In advanced lipedema, chronic inflammation worsens fibrosis, causing subcutaneous tissue and nodules to harden and grow. Damage to lymphatic vessels can result in edema accumulation, leading to lipo-lymphedema. The risk of secondary lymphedema increases in later stages of lipedema. Research suggests that the presence of fibrosis in lymphedematous limbs may limit the effectiveness of conservative treatments such as compression therapy and manual lymphatic drainage (MLD). Fibrotic tissue is less responsive to external compression and manual manipulation, hindering the redistribution of fluid and lymphatic drainage [54]. As a result, individuals with extensive fibrosis may experience slower or less pronounced reductions in limb volume and may be at a higher risk of developing complications such as recurrent infections and skin breakdown. Several studies have investigated the impact of fibrosis on treatment outcomes in lymphedema management. For example, Brorson et al. [53] observed that patients with advanced fibrosis may have limited success with traditional therapies and may require more aggressive interventions such as liposuction or surgical debulking to achieve significant reductions in limb size. Similarly, a study by Lee et al. [56] found that the degree of fibrosis correlated with the severity of lymphedema symptoms and functional impairment, highlighting the importance of assessing fibrotic changes in treatment planning. The extent of fibrosis serves as a valuable predictor for lymphedema treatment outcomes, guiding clinicians in selecting appropriate interventions and managing patient expectations. Further research into strategies for addressing fibrotic tissue and enhancing treatment efficacy in individuals with lymphedema is warranted to improve outcomes and quality of life for affected individuals.

### 3.3. The Presence of Concurrent Conditions

The presence of concurrent conditions can serve as a significant predictor for lymphedema treatment outcomes, influencing response to therapy and overall management strategies. Concurrent conditions, such as obesity, venous insufficiency, or recurrent infections, can impact lymphedema progression, exacerbate symptoms, and complicate treatment efforts. Obesity is commonly associated with lymphedema and can exacerbate swelling and tissue inflammation by placing additional stress on the lymphatic system. Studies have shown that individuals with obesity may have poorer outcomes with conservative treatments such as compression therapy and manual lymphatic drainage (MLD) due to reduced efficacy of compression garments and difficulty accessing lymphatic vessels in dense adipose tissue [57,58,59]. Venous insufficiency, characterized by impaired venous return and chronic venous hypertension, can contribute to lymphedema development or exacerbate existing lymphatic dysfunction. The coexistence of venous insufficiency and lymphedema may complicate treatment planning and require a multidisciplinary approach to address both vascular and lymphatic components [60]. Recurrent infections, such as cellulitis or lymphangitis, are common complications of lymphedema and can lead to acute exacerbations of swelling, pain, and tissue inflammation. The presence of recurrent infections may indicate underlying lymphatic dysfunction and may necessitate aggressive treatment with antibiotics, wound care, and lymphedema management strategies to prevent disease progression and recurrent episodes [61,62]. Assessing and addressing concurrent conditions is essential for optimizing lymphedema treatment outcomes and improving patient quality of life. A comprehensive evaluation that includes a thorough medical history, physical examination, and diagnostic testing is necessary to identify and manage comorbidities effectively. Multidisciplinary collaboration among healthcare providers, including lymphedema specialists, vascular surgeons, infectious disease specialists, and dermatologists, is often required to develop individualized treatment plans tailored to each patient’s unique needs and circumstances.

### 3.4. Compliance and Adherence to Treatment

Compliance and adherence to treatment serve as critical predictors for lymphedema management, significantly influencing treatment outcomes and overall effectiveness. Lymphedema treatment often involves a combination of therapeutic modalities, such as compression therapy, manual lymphatic drainage (MLD), exercise, and skincare, which require consistent and ongoing participation from patients. Research indicates that patient compliance with prescribed treatment regimens is associated with better outcomes in lymphedema management. Adherence to compression garment wear has been shown to correlate with reductions in limb volume, improved lymphatic function, and decreased risk of disease progression [63]. Similarly, regular attendance at MLD sessions and engagement in prescribed exercise programs have been associated with improved symptom control and enhanced quality of life for individuals with lymphedema [64]. Conversely, non-compliance or poor adherence to treatment recommendations can impede treatment success and lead to suboptimal outcomes. Factors contributing to non-compliance may include discomfort or difficulty associated with treatment modalities, lack of awareness or understanding of the importance of treatment, or psychosocial barriers such as depression or anxiety [65]. Non-compliance with compression therapy, for example, has been identified as a significant predictor of recurrent cellulitis and disease exacerbation in patients with lymphedema [66]. Assessing and addressing barriers to compliance and adherence is essential for optimizing treatment outcomes in lymphedema management. Healthcare providers play a crucial role in educating patients about the importance of treatment adherence, addressing concerns or misconceptions, and providing support and encouragement throughout the treatment process. Patient-centered approaches that consider individual preferences, lifestyle factors, and treatment goals can help enhance treatment engagement and improve long-term outcomes for individuals living with lymphedema.

### 3.5. Baseline Limb Volume and Circumference

Baseline limb volume and circumference serve as important predictors for lymphedema management, providing valuable information about disease severity, progression, and treatment response. These baseline measurements serve as benchmarks for assessing changes in limb size over time and evaluating the effectiveness of therapeutic interventions. Several studies have demonstrated the utility of baseline limb volume and circumference measurements as predictors for lymphedema outcomes. For example, a study by Stout- Gergich et al. [37] found that baseline limb volume was significantly associated with treatment response, with larger baseline volumes predicting greater reductions in limb size following Complete Decongestive Therapy (CDT) in patients with breast cancer-related lymphedema [37]. Similarly, baseline limb circumference measurements have been shown to correlate with disease severity and functional impairment in individuals with lymphedema, with larger circumferences indicating more advanced disease and poorer treatment outcomes [67]. In addition to assessing disease severity, baseline limb volume and circumference measurements can help identify individuals at higher risk for lymphedema development or progression. For example, preoperative limb volume measurements have been used to predict the likelihood of developing lymphedema following cancer treatment, allowing for early intervention and preventive measures in high-risk individuals [68]. Furthermore, baseline measurements are essential for individualizing treatment plans and monitoring patient progress over time. By establishing baseline values, healthcare providers can track changes in limb size, assess treatment response, and adjust therapy as needed. Regular monitoring of limb volume and circumference allows for the early detection of disease recurrence or progression, enabling timely intervention to prevent complications and optimize treatment outcomes. Baseline limb volume and circumference measurements are valuable predictors for lymphedema management, providing essential information for treatment planning, monitoring, and optimizing therapeutic interventions.

### 3.6. Age and Body Mass Index (BMI)

Age and body mass index (BMI) are important predictors for lymphedema development, progression, and treatment outcomes. Understanding the impact of age and BMI on lymphedema can help healthcare providers tailor treatment approaches and optimize patient care. Age is a significant predictor for lymphedema risk, with older individuals often being at higher risk due to factors such as decreased tissue elasticity, impaired lymphatic function, and increased comorbidities. Previous studies have shown that advancing age is associated with a higher prevalence of lymphedema, particularly in populations undergoing cancer treatment such as surgery and radiation therapy [69]. Additionally, age-related changes in tissue structure and function may affect the response to lymphedema treatment. Older patients may have slower tissue healing, reduced mobility, and increased susceptibility to complications such as infections. Therefore, age should be considered when developing treatment plans and managing expectations for lymphedema outcomes.

Body mass index (BMI), a measure of body fat based on height and weight, is another important predictor for lymphedema risk and severity. Obesity is a well-established risk factor for lymphedema, as excess adipose tissue can compress lymphatic vessels, impair lymphatic flow, and increase fluid retention [70]. Studies have shown that individuals with higher BMI are more likely to develop lymphedema following cancer treatment, particularly breast cancer-related lymphedema. Higher BMI is also associated with more severe lymphedema symptoms, poorer treatment outcomes, and increased risk of disease progression [63]. Furthermore, obesity can complicate lymphedema management by limiting the effectiveness of compression therapy, increasing the risk of cellulitis and skin breakdown, and exacerbating mobility issues. Therefore, addressing obesity and promoting weight management strategies are important components of comprehensive lymphedema care. In summary, age and BMI are important predictors for lymphedema risk, severity, and treatment outcomes. Healthcare providers should consider these factors when assessing patients for lymphedema, developing treatment plans, and implementing preventive measures.

### 3.7. Lymphedema Etiology

Understanding the etiology of lymphedema is essential for predicting disease progression, identifying individuals at risk, and optimizing treatment outcomes. Lymphedema can arise from various underlying causes, with primary and secondary etiologies playing distinct roles in disease manifestation and management.

#### 3.7.1. Primary Lymphedema

Primary lymphedema is characterized by congenital abnormalities or developmental defects in the lymphatic system, leading to impaired lymphatic drainage and fluid accumulation. Genetic mutations or alterations in lymphatic vessel development can contribute to primary lymphedema, which may be present at birth or later in life. The age of onset and specific genetic factors associated with primary lymphedema can influence disease severity, progression, and treatment response [71].

#### 3.7.2. Secondary Lymphedema

Secondary lymphedema occurs because of the damage, obstruction, or dysfunction of the lymphatic system secondary to external factors such as surgery, radiation therapy, trauma, infection, or underlying medical conditions. Common causes of secondary lymphedema include cancer treatment, particularly surgery and radiation therapy for breast cancer, gynecologic cancers, or melanoma. The extent of lymphatic damage, the presence of comorbidities, and the timing of intervention can all impact the development and progression of secondary lymphedema [72].

Understanding the etiology of lymphedema allows healthcare providers to tailor treatment approaches based on the underlying cause and individual patient characteristics. For example, primary lymphedema may require lifelong management strategies to address congenital lymphatic abnormalities, whereas secondary lymphedema may benefit from early intervention to prevent or minimize lymphatic damage following cancer treatment. Additionally, knowledge of lymphedema etiology can help identify individuals at higher risk for developing the condition and implement preventive measures accordingly. Patients undergoing cancer treatment, lymph node dissection, or radiation therapy should be educated about the risk of lymphedema and encouraged to practice risk-reduction strategies such as skincare, exercise, and avoiding limb constriction.

### 3.8. Psychosocial Factors

Psychosocial factors play a significant role in predicting lymphedema outcomes, influencing treatment adherence, coping strategies, and overall quality of life for individuals living with the condition. Understanding the impact of psychosocial factors can help healthcare providers tailor interventions and support services to address the unique needs of patients with lymphedema. Some of the psychosocial factors are the following:

#### 3.8.1. Emotional Distress

Emotional distress, including anxiety, depression, and fear, is commonly reported among individuals with lymphedema and can significantly impact treatment adherence and disease management. Studies have shown that psychological distress is associated with poorer treatment outcomes, increased symptom burden, and decreased quality of life in patients with lymphedema [69]. Addressing emotional distress through counseling, support groups, and mindfulness-based interventions can help improve coping strategies and enhance treatment engagement.

#### 3.8.2. Body Image Concerns

Body image concerns are prevalent among individuals with lymphedema, particularly those undergoing cancer treatment, surgery, or lymph node dissection. Changes in body appearance, swelling, and alterations in clothing fit can contribute to feelings of self-consciousness, embarrassment, and social isolation. Body image concerns may affect treatment adherence and engagement in social activities, leading to decreased quality of life and psychological well-being [73]. Interventions focusing on body image acceptance, self-esteem enhancement, and adaptive clothing options can help mitigate the impact of body image concerns on lymphedema management.

#### 3.8.3. Social Support

Social support plays a crucial role in predicting lymphedema outcomes, providing individuals with emotional, practical, and informational support to cope with the challenges of living with the condition. Studies have shown that perceived social support is associated with better treatment adherence, improved psychological well-being, and enhanced quality of life in patients with lymphedema [74]. Engaging patients in support groups, peer mentoring programs, and educational workshops can foster social connections, reduce feelings of isolation, and promote resilience in managing lymphedema.

Psychosocial factors serve as important predictors for lymphedema outcomes, influencing treatment adherence, coping strategies, and quality of life. By addressing emotional distress, and body image concerns, and enhancing social support, healthcare providers can optimize treatment engagement and improve overall well-being for individuals living with lymphedema.

### 3.9. Response to Initial Therapy

Response to initial therapy serves as a valuable predictor for lymphedema management, providing insight into treatment effectiveness, disease progression, and the need for additional interventions. The assessment of treatment response allows healthcare providers to tailor ongoing management strategies based on individual patient needs and optimize outcomes.

#### 3.9.1. Complete Decongestive Therapy (CDT)

Complete Decongestive Therapy (CDT) comprises manual lymphatic drainage (MLD), compression therapy, exercise, and skincare, which are the cornerstones of lymphedema management. Response to CDT can vary among patients, with some experiencing significant reductions in limb volume and improvement in symptoms, while others may have limited or partial responses. Studies have shown that early response to CDT is associated with better long-term outcomes, with patients achieving sustained reductions in limb size and improved quality of life [75].

#### 3.9.2. Compression Therapy

Compression therapy, including the use of compression garments, bandaging, or pneumatic compression devices, is commonly employed to reduce limb swelling and maintain lymphatic function. Response to compression therapy can serve as a predictor for treatment outcomes, with patients demonstrating improved compliance, symptom control, and functional status experiencing better long-term results [37].

#### 3.9.3. Exercise

Exercise plays a crucial role in lymphedema management, promoting lymphatic circulation, muscle pump activity, and tissue mobilization. Patients who respond favorably to exercise therapy typically experience reductions in limb volume, improved range of motion, and enhanced functional capacity. Response to exercise can serve as an indicator of treatment adherence and overall treatment success [76].

Assessing response to initial therapy allows healthcare providers to monitor treatment progress, identify barriers to improvement, and make timely adjustments to treatment plans. Lack of response or inadequate improvement may prompt the consideration of alternative treatment modalities, such as surgical interventions or advanced lymphedema therapies, to achieve optimal outcomes.

## 4. Influence of Skin Thickness on Success of Decompressive Therapy of Lymphedema

Skin thickness can indeed play a significant role in the success of decompressive therapy for lymphedema. Lymphedema, characterized by the accumulation of protein-rich fluid in interstitial spaces due to impaired lymphatic drainage, often leads to tissue fibrosis and thickening of the skin over time. Skin changes in lymphedema include fibrosis, hyperkeratosis, and increased thickness, which can further compromise lymphatic function and exacerbate fluid accumulation. The effectiveness of decompressive therapy, such as manual lymphatic drainage (MLD), compression bandaging, and pneumatic compression devices, in managing lymphedema depends partly on the condition of the skin. Thickened skin can impede the efficacy of these therapies by reducing the ability of the lymphatic vessels to respond to external pressure and by limiting the mobility of underlying tissues. Studies have demonstrated that individuals with thicker skin due to lymphedema may experience poorer outcomes with standard compression therapy compared to those with thinner skin. For instance, a study by Moffatt et al. [77] found that patients with thicker skin in chronic lymphedema had reduced response rates to compression therapy. Similarly, a study by Devoogdt et al. indicated that skin thickness was negatively correlated with the effectiveness of MLD in reducing limb volume in breast cancer-related lymphedema. Moreover, the presence of fibrotic tissue and increased skin thickness may necessitate modifications to conventional treatment approaches. Techniques such as scar tissue mobilization, deep tissue massage, or use of specialized bandaging materials may be required to address fibrosis and improve lymphatic drainage in individuals with thicker skin [78]. Therefore, while decompressive therapies remain integral to the management of lymphedema, the influence of skin thickness on treatment outcomes cannot be overlooked. Clinicians should consider skin characteristics, including thickness and fibrosis, when designing individualized treatment plans for patients with lymphedema. In this review, we will discuss some of the ways how skin thickness influences the success of decompressive therapy.

### 4.1. Ease of Application, Tissue Compliance, and Risk of Complications

The ease of applying decompressive therapy on thickened skin significantly affects lymphedema management. Thickened skin, common in chronic lymphedema, obstructs lymphatic vessels and alters compression force distribution, reducing therapy effectiveness in promoting lymphatic drainage and reducing swelling [37,79,80]. It also limits tissue mobility, leading to uneven pressure, discomfort, and decreased efficacy, which may deter patient adherence [81]. To address these challenges, clinicians can use pre-treatment strategies like skin moisturization, softening agents, or gentle massage to improve pliability. Specialized bandaging techniques, multi-layer systems, custom garments, or pneumatic compression devices can enhance compression distribution [79,80,81]. Patient’s education on application techniques, skincare, and adherence is essential to optimize outcomes. By improving application on thickened skin, decompressive therapy becomes more effective, enhancing patient comfort and promoting long-term adherence [37,79,80,81].

Skin thickness significantly affects tissue compliance and the success of decompressive therapy for lymphedema. Thicker skin reduces pliability and elasticity, requiring higher compression pressures for effective lymphatic drainage and fluid reduction. Inadequate compression due to skin thickness can lead to suboptimal outcomes [82]. Thick skin also increases resistance to manual lymphatic drainage (MLD), making lymph mobilization challenging and potentially causing discomfort, irritation, or injury, which affects adherence and efficacy [83,84]. Tailoring therapy to skin thickness involves adjusting compression garments, bandaging techniques, and MLD protocols to ensure effective tissue mobilization and lymphatic drainage. Strategies like soft tissue mobilization, scar massage, and skin hydration can improve compliance and outcomes [36]. Regular assessments of skin thickness provide insights into changes in pliability and lymphatic function, guiding therapy adjustments. Multidisciplinary collaboration among lymphedema specialists, physiotherapists, and dermatologists is essential for addressing skin-related concerns and optimizing care [85]. Tailored approaches improve treatment outcomes and enhance the quality of life for individuals with lymphedema.

Skin thickness significantly impacts decompressive therapy for lymphedema, influencing outcomes and complications. Thickened skin is more prone to irritation, chafing, and damage from poorly fitting or prolonged use of compression garments, leading to skin breakdown, ulceration, or dermatitis, which can reduce adherence and efficacy [25]. Impaired barrier function, combined with compromised lymphatic drainage and immune function, increases susceptibility to infections like cellulitis and lymphangitis, requiring prompt treatment and potentially disrupting therapy [86]. Thicker skin also delays wound healing in cases of skin breakdown due to reduced perfusion, oxygenation, and immune function, prolonging recovery and hindering progress. Tailored wound care strategies are critical to support healing and prevent complications [87]. Additionally, thickened skin may cause discomfort, restrict movement, or make compression garments harder to use, reducing compliance with therapy recommendations. Optimizing garment fit and providing supportive care can enhance adherence and comfort [88,89]. Healthcare providers should assess skin characteristics, monitor for complications, and implement preventive measures to minimize risks and improve treatment outcomes.

### 4.2. Lymphatic Drainage and Response to Treatment

Skin thickness significantly impacts the success of decompressive therapy for lymphedema by influencing lymphatic drainage and fluid dynamics, critical for reducing limb swelling. Thicker skin may resist lymphatic flow, impair drainage, and reduce the effectiveness of therapy in alleviating swelling and improving tissue health [60]. Compression therapy, a cornerstone of lymphedema management, relies on external pressure to promote lymphatic uptake and fluid movement. Thicker skin can impede compression forces from reaching deeper tissues, limiting its efficacy [90]. Similarly, manual lymphatic drainage (MLD) aims to enhance lymphatic transport, but thicker skin presents resistance, requiring a precise technique and adequate pressure for effective results [91]. Adjunct therapies like pneumatic compression or low-level laser therapy can complement decompressive therapy by promoting lymphatic drainage and reducing fibrosis. However, their effectiveness may vary with skin thickness and tissue compliance, emphasizing the need for individualized approaches [92]. Understanding how skin thickness affects lymphatic function is essential to optimize treatment and improve quality of life for individuals with lymphedema.

The response to lymphedema treatment is influenced by various factors, including skin thickness, which can affect tissue compliance, lymphatic drainage, and adherence to decompressive therapy. Thicker skin may cause discomfort, restricted mobility, or difficulty with compression garments, reducing adherence and treatment efficacy [25,60]. Effective lymphatic drainage is essential for reducing limb swelling, and tissue compliance plays a role in mobilizing lymph fluid. Monitoring treatment response, such as limb volume reduction and symptom improvement, helps providers adjust interventions and optimize outcomes [93,94]. However, evidence suggests that skin thickness does not significantly impact the success of decompressive therapy. Studies by Perez et al. [95], Yoshida et al. [96], and Forte et al. [97] found that success is more influenced by postoperative compression, treatment continuity, and adherence than by skin thickness. Lanza et al. [50] and Can et al. [46] highlighted that factors like treatment frequency, proper compression application, and adherence are more critical. Tidhar et al. [98] emphasized that self-management and adherence to prescribed therapies significantly reduce edema, regardless of skin thickness. While skin thickness is a consideration, it is just one of many factors in lymphedema management. Individualized assessment of skin condition, tissue characteristics, and overall health is essential to tailor treatment plans. Continuous monitoring and adjustments help to address changes in skin thickness and other factors over time to optimize patient outcomes (Table 2 and Table 3).

## 5. Effect of Fat Layer Thickness on Success of Decompressive Therapy of Lymphedema

The thickness of the fat layer of the skin can indeed influence the success of decompressive therapy for lymphedema.

### 5.1. Impact on Compression Therapy and Role in Lymphatic Drainage

The thickness of the fat layer impacts the effectiveness of compression therapy in lymphedema by influencing pressure distribution, lymphatic transport, tissue compliance, and treatment adherence. Thicker fat layers make achieving adequate pressure for lymphatic drainage and swelling reduction more challenging, often requiring customized compression garments or specialized bandaging techniques [90,99]. Compression therapy relies on consistent pressure to promote lymphatic transport by facilitating fluid uptake and movement toward lymphatic collectors. Thicker fat layers can impede flow, requiring tailored compression protocols to optimize outcomes [100]. Reduced tissue compliance in thicker fat layers may also hinder effective compression and cause discomfort, necessitating adjustments to garment selection and compression parameters to maintain efficacy and mobility [2].

Treatment adherence is crucial for compression therapy success. Individuals with thicker fat layers may face difficulties with compression garments, reducing compliance. Providing education, support, and customized solutions can enhance adherence and improve outcomes [101]. Addressing adipose tissue thickness in compression protocols is essential to optimize pressure distribution, lymphatic function, and treatment adherence, ultimately improving quality of life for individuals with lymphedema.

The thickness of the fat layer impacts tissue compliance, lymphatic drainage, and fluid distribution in lymphedema management. Excessive adipose tissue compresses lymphatic vessels, impedes lymphatic flow, and exacerbates fluid accumulation, contributing to lymphedema progression [60,101]. Thicker fat layers create challenges for compression therapy by reducing the ability of garments or bandages to exert sufficient pressure, resulting in uneven fluid distribution and suboptimal outcomes [102]. Adipose tissue thickness affects tissue compliance, limiting the ability to redistribute fluid effectively and contributing to persistent swelling, fibrosis, and functional impairment [103]. Addressing these challenges requires tailoring treatments to individual patient characteristics. Strategies like manual lymphatic drainage (MLD), pneumatic compression therapy, and adjunctive modalities can enhance lymphatic drainage and treatment efficacy [104]. In summary, fat layer thickness influences lymphatic drainage, compression therapy, and tissue compliance. Assessing and addressing adipose tissue thickness is crucial for optimizing treatment strategies and improving outcomes for individuals with lymphedema.

### 5.2. Challenges in Manual Techniques, Surgical Considerations, and Adherence to Treatment

Adipose tissue thickness can hinder the effectiveness of manual lymphatic drainage (MLD), limiting penetration depth and fluid mobilization. Thicker fat layers reduce the transmission of manual pressure to lymphatic vessels, requiring modifications in MLD protocols or the use of adjunct therapies to enhance lymphatic transport [105,106,107]. Increased fat layer thickness may also necessitate longer treatment durations or more frequent sessions for comparable outcomes. Tailoring MLD techniques to individual adipose tissue characteristics improves treatment efficacy, symptom relief, and functional outcomes [108,109]. Providers should assess tissue thickness and adapt protocols, combining MLD with customized compression and adjunct therapies for optimal results [110].

Thicker fat layers affect surgical feasibility in lymphedema management by increasing challenges in tissue exposure and lymphatic vessel identification. Procedures such as lymphatic venous anastomosis (LVA) or lymph node transfer may be less effective due to difficulty accessing target structures [111]. Adipose tissue thickness also elevates risks of postoperative complications like wound healing issues, infection, and tissue necrosis, requiring careful preoperative assessment and individualized surgical planning [112]. Patient education on surgical options, benefits, and risks is vital to ensure informed decision making and satisfaction with outcomes [41,113].

Thicker fat layers may complicate the use of compression garments, making them harder to don and potentially causing discomfort or skin irritation. These challenges can reduce adherence to prescribed treatments and compromise outcomes [114,115]. Limited mobility and self-care difficulties, such as skincare and bandaging, further hinder treatment adherence. Psychological factors like negative body image and emotional distress may also influence compliance, emphasizing the need for psychosocial support alongside physical interventions [74,116,117].

Adipose tissue thickness impacts MLD efficacy, surgical feasibility, and treatment adherence in lymphedema management. Healthcare providers should adapt treatment strategies—including compression therapy, manual techniques, surgical interventions, and psychosocial support—based on patient-specific characteristics. Tailored approaches can overcome these challenges, optimizing lymphatic drainage, improving patient satisfaction, and enhancing overall outcomes (Table 2 and Table 3).

## 6. Follow-Up and Preventive Strategies

Both primary and secondary lymphedema are chronic conditions requiring continuous monitoring and management. Here, we will discuss the follow-up and preventive strategies of therapies that are commonly administered to or undertaken by lymphoedema patients.

Studies indicate that limb volume reduction and/or a decrease in percentage edema can be achieved through standard Complete Physical Therapy (CPT), CPT combined with pump therapy, or a combination of therapies. These methods demonstrated continued volume reduction at follow-up intervals ranging from 1 to 12 months [118,119,120,121]. However, none of the studies reported whether these improvements were consistently sustained over time. The optimal treatment period for CPT appears to be one month, though two studies [119,122] observed volume reduction after just 7–8 days of treatment. Patients who received manual lymphatic drainage (MLD) experienced a volume reduction ranging from 104 to 156 mL (8–10%), with one study reporting sustained volume reduction at six months post-treatment [118]. Compression therapy also showed efficacy, with volume reductions of 38 mL (7%) [118] and 20 mL (4% edema) [123], along with significant improvements in sensations of arm heaviness and tension. Two studies evaluated the effects of 30–40 mmHg compression garments. One study with 12 participants who wore the garment for two weeks [124] reported a significant volume reduction of 49 mL (~5%) and improvements in tension and heaviness. Another study with 26 participants wearing compression garments for six months [125] reported similar benefits. Exercise regimens were shown to have varied effects on limb volume, ranging from reductions of 12 to 101 mL (0.4–9%), with three studies noting improvements in subjective symptoms [118,126]. Sustained reductions were observed between 24 h and six weeks after the cessation of exercise programs [118,126].

Clinical follow-ups should be scheduled every six months, though complications may necessitate closer monitoring. Each visit should include a physical examination, circumferential measurements, and weight control assessments. Pressure garments need replacement every 6–9 months. Self-management practices, exercise adherence, and patient or family compliance should also be reviewed.

Prevention is classified into primary and secondary preventions. Primary prevention is for individuals at risk; this includes maintaining a healthy weight, proper skincare, regular exercise, and using low-pressure garments during flights or avoiding strenuous activities [2,127,128]. Secondary prevention is aimed at preventing complications like cellulitis; it emphasizes proper skincare, consistent use of pressure garments, and self-management to prevent worsening edema and other complications [127,128]. Lymphedema is a chronic condition requiring lifelong, multidisciplinary care under PMR specialists. These specialists play a vital role in the diagnosis, treatment, and personalized management strategies to improve patient outcomes and quality of life. While self-maintenance therapies show modest improvements, they are more effective than no treatment and beneficial when professional care is unavailable. Ongoing therapy is essential to maintain improvements, highlighting the chronic nature of the disease. Larger, high-quality clinical trials are needed to strengthen evidence for these treatments.

## 7. Conclusions

Lymphedema, a debilitating condition marked by the accumulation of lymphatic fluid and subsequent swelling, can significantly impair the quality of life and functional capacity of an individual. The comprehensive management of lymphedema through decongestive therapy, which combines manual lymphatic drainage, compression therapy, skincare, and exercise, is essential for mitigating the physical and psychological burdens of the disease.

The efficacy of decongestive therapy largely hinges on several critical factors. An early diagnosis and the timely initiation of therapy play pivotal roles in preventing the progression of lymphedema to its more severe, often irreversible stages. Adherence to a meticulously tailored therapeutic regimen that includes all components of Complete Decongestive Therapy is crucial for achieving optimal outcomes.

Moreover, recent research has illuminated the significant influence of both skin and adipose tissue characteristics on the success of decongestive therapy. Increased skin thickness and a higher volume of adipose tissue have been identified as factors that can diminish the effectiveness of traditional decongestive methods, necessitating adjustments in therapy that may include more advanced techniques such as surgical interventions or specialized compression protocols.

In conclusion, managing lymphedema effectively requires a nuanced approach that considers the individual characteristics of the lymphedema and the patient. Successful outcomes are most likely when therapy is personalized, initiated early, and diligently followed, with adaptations made as necessary to address the specific challenges posed by the physical properties of affected tissues. Continued research into the predictors of theraFpeutic success and the impacts of tissue characteristics will further enhance our ability to provide targeted and effective interventions for those suffering from lymphedema.

## Figures and Tables

**Table 1:** Key factors and predictors for the success in Lymphedema Management.

Categories	Predictors	Details
Patient and Disease Characteristics	Stage and Severity	Early-stage lymphedema responds better; advanced stages with fibrosis need aggressive interventions.
	Extent of Fibrosis	Limits the effectiveness of conservative treatments; may require surgical interventions like liposuction.
	Age	Older age correlates with higher lymphedema risk and slower treatment response.
	BMI	Higher BMI increases swelling, worsens outcomes, and complicates compression therapy.
	Lymphedema Etiology	Primary: congenital defects; secondary: caused by treatments like surgery and radiation.
Interventions and Protocols	Compression Therapy	Consistent use reduces limb volume and improves outcomes.
	Manual Lymphatic Drainage (MLD)	Effective in early stages; less effective in advanced fibrosis cases.
	Tailored Interventions	Individualized compression and exercise plans enhance success.
	Early Intervention	Immediate postop therapy reduces lymphedema volume effectively.
	Diagnostic Tools	Lymphoscintigraphy helps predict therapy response and plan interventions.
Adherence and Support	Patient Adherence	Consistent adherence to compression, MLD, and exercise ensures better outcomes.
	Social Support	Emotional and practical support fosters better treatment engagement.
	Psychosocial Factors	Addressing emotional distress and body image issues improves adherence and quality of life.
Measurement and Monitoring	Baseline Measurements	Initial limb volume and circumference help predict treatment outcomes and progression.
	Response to Initial Therapy	Early improvements predict long-term success.

**Table 2:** Advantages and disadvantages of treatment choices in lymphedema management.

Treatment Choices	Advantages	Disadvantages
Manual Lymphatic Drainage (MLD)	- Reduces limb volume and swelling. - Improves lymphatic flow and quality of life. - Gentle and non-invasive.	- Time-consuming. - Requires trained therapists. - Less effective for severe fibrosis.
Compression Therapy	- Promotes lymphatic drainage. - Reduces limb volume. - Maintains post-reduction size.	- Can cause discomfort or irritation. - May require customization for effectiveness.
Exercise	- Enhances lymphatic flow. - Improves mobility and physical function. - Supports weight management.	- Needs careful supervision to avoid strain. - May not be suitable for all stages.
Pneumatic Compression Therapy (PCT)	- Facilitates lymphatic drainage. - Reduces swelling and tissue fluid content.	- Expensive equipment. - Requires regular and correct usage.
Complete Decongestive Therapy (CDT)	- Combines multiple approaches for holistic care. - Effective for various stages.	- Resource-intensive. - Requires patient adherence.
Surgical Interventions	- Effective for advanced cases. - Can significantly reduce limb volume.	- Invasive and carries surgical risks. - Requires post-surgical compression and care.
Liposuction	- Removes excess fat and improves mobility. - Effective for late-stage lymphedema.	- Risk of complications. - Not a standalone treatment.
Adjunctive Therapies	- Complements other therapies. - Reduces tissue fibrosis.	- Effectiveness may vary. - Requires additional resources.

**Table 3:** Summary of complications associated with each treatment method for lymphedema.

Treatment Methods	Complications
Manual Lymphatic Drainage (MLD)	- Skin irritation due to excessive pressure - Reduced effectiveness in patients with fibrosis or thickened skin - Discomfort in individuals with limited tissue compliance
Compression Therapy	- Pressure sores and skin irritation from improper garment fit - Discomfort or restricted mobility - Reduced adherence due to garment application challenges
Exercise	- Potential injury from unsupervised or improper techniques - Exacerbation of symptoms in high-intensity exercises
Pneumatic Compression Therapy (PCT)	- Discomfort from device use - Limited effectiveness in advanced fibrosis - Difficulty in achieving patient adherence
Complete Decongestive Therapy (CDT)	- Requires patient adherence to all components - Challenges with fibrosis or advanced tissue damage reduce effectiveness
Surgical Interventions	- Risk of wound healing complications - Postoperative infections or tissue necrosis - Challenges in lymphatic structure access with thicker fat layers
Skin and Adipose Tissue Modifications	- Challenges in treating areas with fibrosis or excessive fat layers - Risk of uneven compression in affected areas
Adherence-related Challenges	- Reduced effectiveness due to poor adherence - Psychological distress and body image concerns affect consistency
